# Percutaneous Coronary Intervention Utilization and Appropriateness across the United States

**DOI:** 10.1371/journal.pone.0138251

**Published:** 2015-09-17

**Authors:** Michael P. Thomas, Craig S. Parzynski, Jeptha P. Curtis, Milan Seth, Brahmajee K. Nallamothu, Paul S. Chan, John A. Spertus, Manesh R. Patel, Steven M. Bradley, Hitinder S. Gurm

**Affiliations:** 1 Cardiovascular Medicine, University of Michigan, Ann Arbor, Michigan, United States of America; 2 Cardiovascular Medicine, VA Ann Arbor Healthcare System, Ann Arbor, Michigan, United States of America; 3 Cardiovascular Medicine, Yale School of Medicine, New Haven, Connecticut, United States of America; 4 Saint Luke’s Mid America Heart and Vascular Institute, Kansas City, Missouri, United States of America; 5 Duke Clinical Research Institute, Durham, North Carolina, United States of America; 6 Cardiovascular Medicine, University of Colorado and VA Eastern Colorado Healthcare System, Denver, Colorado, United States of America; Cliniche Humanitas Gavazzeni, ITALY

## Abstract

**Background:**

Substantial geographic variation exists in percutaneous coronary intervention (PCI) use across the United States. It is unclear the extent to which high PCI utilization can be explained by PCI for inappropriate indications. The objective of this study was to examine the relationship between PCI rates across regional healthcare markets utilizing hospital referral regions (HRRs) and PCI appropriateness.

**Methods:**

The number of PCI procedures in each HRR was obtained from the 2010 100% Medicare limited data set. HRRs were divided into quintiles of PCI utilization with increasing rates of utilization progressing to quintile 5. NCDR CathPCI Registry^®^ data were used to evaluate patient characteristics, appropriate use criteria (AUC), and outcomes across the HRR quintiles defined by PCI utilization with the study population restricted to HRRs where ≥ 80% of the PCIs were performed at institutions participating in the registry. PCI appropriateness was defined using 2012 AUC by the American College of Cardiology (ACC)/American Heart Association (AHA)/The Society for Cardiovascular Angiography and Interventions (SCAI).

**Results:**

Our study cohort comprised of 380,981 patients treated at 178 HRRs. Mean PCI rates per 1,000 increased from 4.6 in Quintile 1 to 10.8 in Quintile 5. The proportion of non-acute PCIs was 27.7% in Quintile 1 increasing to 30.7% in Quintile 5. Significant variation (p < 0.001) existed across the quintiles in the categorization of appropriateness across HRRs of utilization with more appropriate PCI in lower utilization areas (Appropriate: Q1, 76.53%, Q2, 75.326%, Q3, 75.23%, Q4, 73.95%, Q5, 72.768%; Inappropriate: Q1 3.92%, Q2 4.23%, Q3 4.32%, Q4 4.35%, Q5 4.05%; Uncertain: Q1 8.29%, Q2 8.84%, Q3 8.08%, Q4 9.01%, Q5 8.93%; Not Mappable: Q1 11.26%, Q2 11.67%, Q3 12.37%, Q4 12.69%, Q5 14.34%). There was no difference in risk-adjusted mortality across quintiles of PCI utilization.

**Conclusions:**

Geographic regions with lower PCI rates have a higher proportion of PCIs performed for appropriate indications. Areas that perform more PCIs also appear to perform more elective PCI and many could not be mapped by the AUC.

## Introduction

Percutaneous coronary intervention (PCI) is one of the most commonly performed procedures in the United States and is a major contributor to health care cost. Prior studies have reported a 5-fold variation in rates of PCI across geographic regions. [1] As much of this geographic variation is attributable to differences in rates of PCI for non-urgent indications, it is possible that practice variation is a major driver of this dissimilarity. However, it is unknown the extent to which geographic variation in PCI procedures is due to inappropriate use of PCI.

The American College of Cardiology (ACC), American Heart Association (AHA), and The Society for Cardiovascular Angiography and Interventions (SCAI) have developed well-established guidelines for the performance of PCI. [2] In 2009, a multi–society effort resulted in the development of Appropriate Use Criteria (AUC) for Coronary Revascularization [3]. These criteria serve as a standardized tool to assess the likelihood of clinical benefit from a given PCI procedure. Initial studies have indicated that almost all PCIs performed for acute indications are appropriate whereas the rate of inappropriate PCI (in which the expected benefit of treatment is small) is between 12% to 20% for non-acute indications. [4–7]

The objective of this study was to better understand the overall relationship between utilization of PCI across regional healthcare markets across the United States and appropriateness. We hypothesized that previously described geographic variations in PCI rates may be driven by different patterns of use of non-urgent, inappropriate procedures, with little geographic variation in appropriate PCI.

This research was supported by the American College of Cardiology Foundation’s National Cardiovascular Data Registry (NCDR). The views expressed in this manuscript represent those of the authors, and not necessarily represent the official views of the NCDR or its associated professional societies identified at www.ncdr.com.

## Methods

### Funding and Content

No extramural funding was used to support this work. The authors are solely responsible for the design and conduct of this study, all study analyses, the drafting and editing of the paper and its final contents.

### CathPCI Registry

Our study was comprised of patients undergoing PCI in 2010 at hospitals participating in the NCDR CathPCI Registry. The details and design of the NCDR CathPCI Registry, a joint initiative of the American College of Cardiology and the Society for Cardiovascular Angiography and Interventions, have been described previously. [8,9] Briefly, the registry assesses characteristics, treatments, and in-hospital outcomes of patients undergoing diagnostic catheterization or percutaneous intervention at over 1,500 United States sites. The data are collected by trained staff at participating hospitals using standardized data elements. The data collection process, audit and quality checks have been previously described. Waiver of written informed consent and authorization for this study was granted by Chesapeake Research Review Incorporated. Patient records and information was anonymized and de-identified prior to analysis.

### Categorizing Procedural Appropriateness

The methodology for the appropriateness criteria for coronary revascularization has been described previously. [3] Briefly, an expert panel from various specialties and subspecialties reviewed a set of 198 prototypical clinical scenarios and adjudicated whether or not the literature and clinical experience supported that the benefits of the procedure, in terms of mortality reduction or symptom relief, outweighed the costs and risks. Each clinical scenario was scored on a numeric scale from 1 indicating the least appropriate and 9 suggesting the most. Scores were further categorized into ratings of appropriate (median score = 7–9), uncertain (4–6), or inappropriate (1–3). The terminology was changed in 2013 to appropriate care, may be appropriate care, and rarely appropriate care; however, the aforementioned terminology was utilized given the application of the 2012 ACC/AHA/SCAI AUC in the current work. [10] Appropriate coronary revascularization is considered generally acceptable and a reasonable approach for the indication that is likely to improve the patients’ outcomes and survival while coronary revascularization is considered inappropriate when it is not generally acceptable and not a reasonable approach for the indication as it is unlikely to improve the patients’ outcomes and survival. Coronary revascularization is considered uncertain when more research or patient information is required to classify as appropriate or inappropriate. Previous work demonstrated minimal rates of inappropriate PCIs in patients being treated for an acute coronary syndrome (ACS), but almost 1 in 8 procedures in the setting of stable coronary artery disease (CAD) were inappropriate and nearly 1 in 6 PCIs were unmappable due to missing data. [4]

### Statistical Analysis

#### Assessing geographical variation in PCI

Geographical variation was assessed by dividing the country into hospital referral regions (HRRs). An HRR is a regional health care market for tertiary care that contains at least one hospital performing major cardiovascular procedures and neurosurgery. [11] The number of PCI procedures in each zip code was obtained by identifying claims with relevant PCI ICD-9 and CPT procedure codes from the 100% Medicare limited data set from 2010 (please see **S1Table**for relevant codes). These data were then aggregated to the HRR level by mapping patient zip code to HRR using zipcode crosswalk files from the Dartmouth Atlas of Health Care (http://www.dartmouthatlas.org/tools/downloads.aspx#crosswalks). The rate of PCI use was calculated at the HRR level by dividing the number of PCIs performed on Fee For Service (FFS) Medicare Patients by the total number of eligible FFS Medicare patients in that HRR. We then divided HRRs into quintiles of PCI utilization with quintile 5 representing the highest utilization rate.

All PCIs (irrespective of payer status) in the NCDR CathPCI registry were then assigned to the respective HRRs and quintiles of utilization (derived using Medicare data) using patient zip code. To avoid misclassification due to incomplete participation in the CathPCI registry, we restricted the analyses to those HRRs in which greater than 80% of the total number of PCIs were performed at hospitals participating in the CathPCI Registry.

Data from the NCDR CathPCI Registry were then used to evaluate patient characteristics, procedural appropriateness, and in-hospital outcomes across the quintiles of PCI utilization. Appropriateness was defined using the 2012 ACC/AHA/SCAI AUC and PCIs were categorized as appropriate, inappropriate, uncertain, or unmappable. Unmappable cases are those in which no stress test was performed and the presentation was non-ACS or for a non-ACS presentation with a positive stress test and an unavailable result. [3,12]

#### Assessing variation in appropriateness

The difference in appropriateness across quintiles of PCI utilization was compared using a Chi square test. Additionally, a Cochran-Armitage trend test was performed on each level of the appropriate use criteria. To assess for differences in outcome across the areas of varying PCI utilization, observed and adjusted mortality rates across quintiles were calculated. The NCDR mortality risk model was used to adjust for differences in patient mix. [13] The adjusted rates represented the indirect standardized mortality ratio (observed over expected number of deaths) multiplied by the observed overall rate.

The analysis was repeated utilizing only those HRRs with 100% capture (i.e. all PCIs in that HRR were performed at hospitals participating in the CathPCI Registry).

## Results

### Patient Population

**Fig 1:**
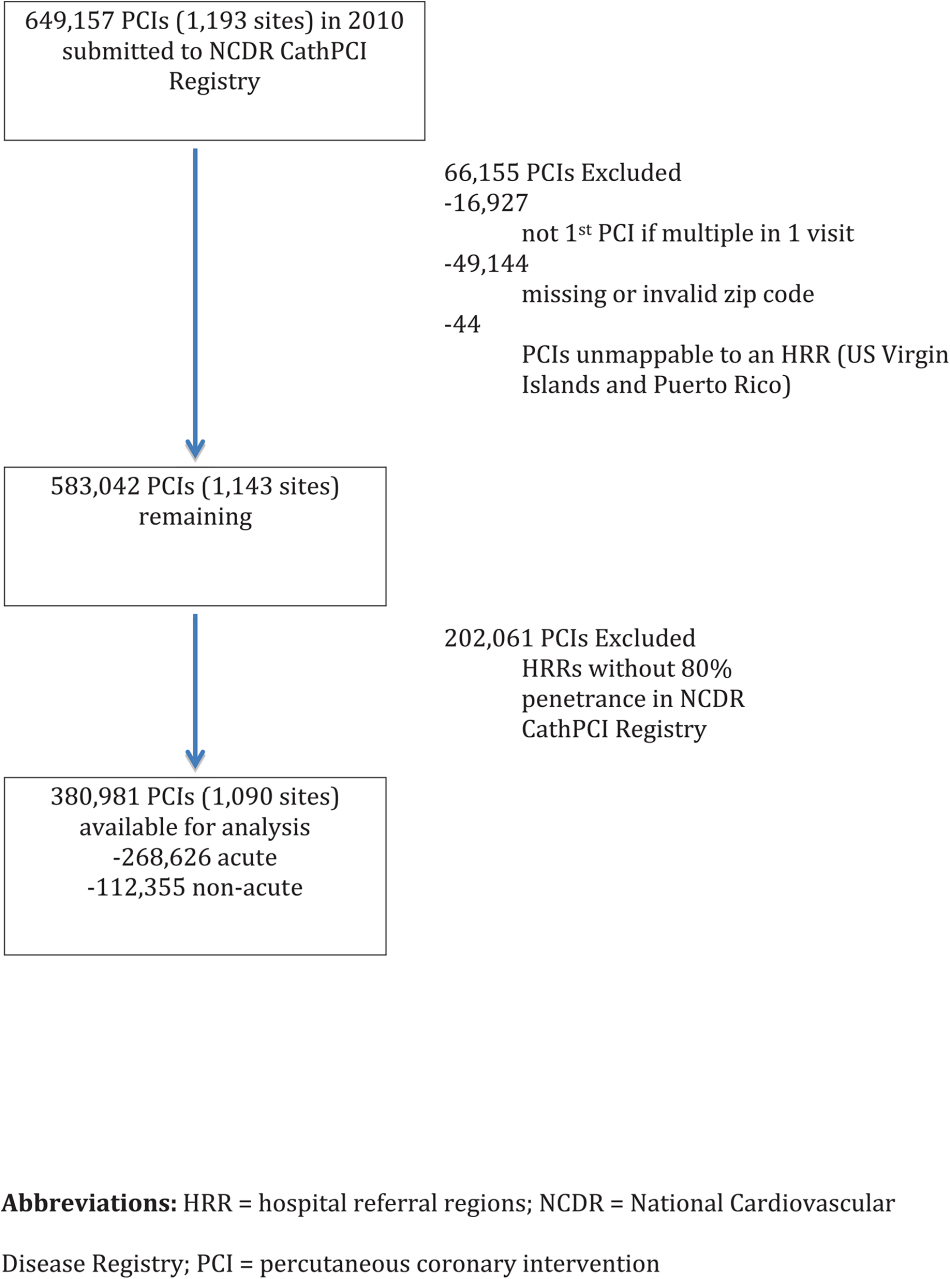
Final cohort available for primary analysis. Shown is the final cohort for the primary analysis and the PCIs that were excluded.

The study population selection is outlined in **Fig 1**. Of the 306 HRRs spanning the United States, 178 were included in the analysis. Of the 178 HRRs included, 100 HRRs had 100% capture (i.e. all sites within that HRR participating in the NCDR CathPCI Registry) while 78 HRRs had 80–99.9% capture. A total of 380,981 patients underwent their first PCI in 2010 and had a valid zip code that could be mapped to these 178 HRRs with ≥80% penetrance in the NCDR.

**Fig 2:**
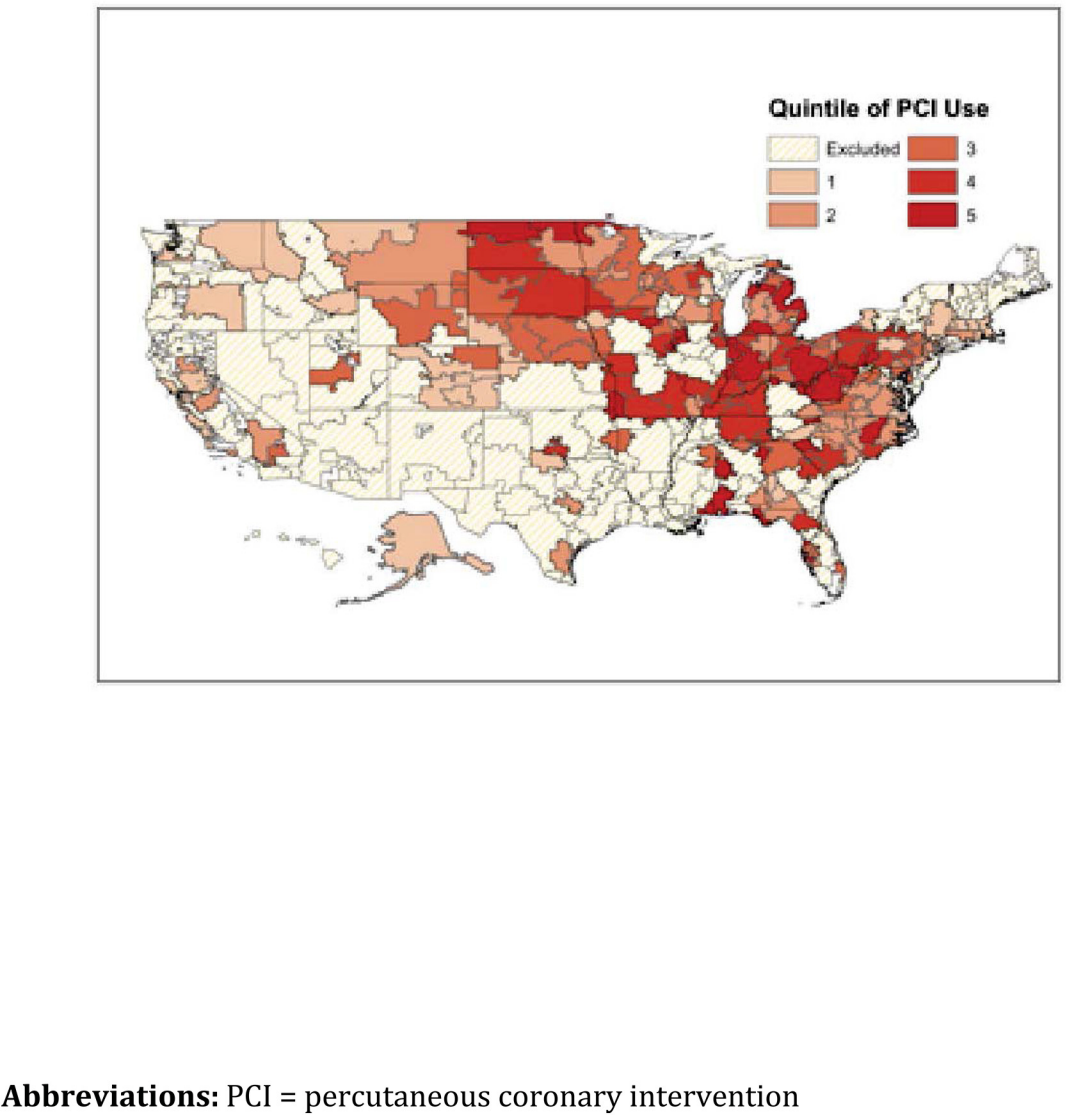
Geographic distribution of HRRs categorized by quintiles of PCI utilization and included in analysis. Shown are the HRRs divided by quintile of PCI utilization and the HRRs that were excluded from the analysis.

The extent of geographic variation in PCI rates for HRRs included in the analysis is depicted in **Fig 2**and for the entire country in **S1 Fig**. The mean PCI rate per 1000 progressing from Quintile 1 to Quintile 5 is 4.6, 6.0, 6.9, 8, and 10.8, respectively (**Table 1)**

**Table 1:** Mean PCI rates within each quintile of utilization (per 1000).

Quintile	# of HRRs	Mean % Capture	Mean PCI Rate (SD)
1	37	98.6%	4.6 (.61)
2	40	96.7%	6.0 (.31)
3	37	96.9%	6.9 (.27)
4	30	95.1%	8.0 (.42)
5	34	97%	10.8 (2.0)

**Abbreviations:** HRR = hospital referral regions; PCI = percutaneous coronary intervention

### PCI Distribution and Patient Characteristics

The average age of the total cohort was 64.5 years and 67.1% were male. 28.6% of the population were current smokers or had smoked within the past year and over 80% of the population had either hypertension or dyslipidemia, or both. Diabetes was present in 35.6% of the cohort. Of all the PCIs, 14.09% were for ST-elevation myocardial infarction (STEMI), 44.93% for non-STEMI or unstable angina, and 41% were for stable CAD, with 9.27% of the cohort being asymptomatic at the time of intervention. **Tables 2 and 3**summarizes the patient demographic and clinical characteristics stratified by quintile of PCI rate. In Quintile 1, of the 55,727 PCIs performed, 72.3% were for ACS compared to Quintile 5 where 69.3% of the 80,010 PCIs were for ACS (proportion of patients who underwent PCI for ACS in quintiles 2,3, and 4 are 70.6%, 71.5%, and 69%, respectively).

**Table 2:** Baseline demographic characteristics of all patients stratified by quintile of PCI rate.

Variable	Total #,%	Quintile 1 #,%	Quintile 2 #,%	Quintile 3 #,%	Quintile 4 #,%	Quintile 5 #,%
Total	380981, 100	55727, 100	72942, 100	88886, 100	83416, 100	80010, 100
Age Mean (SD)	64.5, 12.2	64.5, 12.1	64.4, 12.2	64.8, 12.3	64.4, 12.2	64.5, 12.1
Male	255693, 67.1	39051, 70.1	49703, 68.1	59823, 67.3	55316, 66.3	51800, 64.7
White Race	341206, 89.6	49172, 88.2	63615, 87.2	79543, 89.5	76658, 91.9	72218, 90.3
Private Insurance	244820, 64.3	36012, 64.6	47037, 64.5	58442, 65.8	52345, 62.8	50984, 63.7
Public Insurance	217889, 57.2	30120, 54.1	41152, 56.4	49675, 55.9	47988, 57.5	48954, 61.2
Non-US Insurance	137, 0.04	20, 0.04	24, 0.03	59, 0.07	19, 0.02	15, 0.02
No Insurance	23817, 6.3	3070, 5.5	4934, 6.8	5734, 6.5	5350, 6.4	4729, 5.9

**Table 3:** Baseline clinical characteristics of all patients stratified by quintile of PCI rate.

Variable		Total #,%	Quintile 1 #,%	Quintile 2 #,%	Quintile 3 #,%	Quintile 4 #,%	Quintile 5 #,%
Total		380981, 100	55727, 100	72942, 100	88886, 100	83416, 100	80010, 100
Current / Recent Smoker (w/in 1 year)		108719, 28.6	14256, 25.6	20816, 28.6	24227, 27.3	25349, 30.4	24071, 30.1
Hypertension		311080, 81.7	44082, 79.1	58911, 80.8	72158, 81.2	68230, 81.9	67699, 84.7
Dyslipidemia		307237, 80.7	44379, 79.7	58068, 79.7	72534, 81.7	67152, 80.6	65104, 81.5
Family History of Premature CAD		97210, 25.5	12745, 22.9	19586, 26.9	21481, 24.2	24056, 28.9	19342, 24.2
Prior MI		116895, 30.7	15811, 28.4	21963, 30.1	27247, 30.7	25708, 30.8	26166, 32.7
Prior Heart Failure		44787, 11.8	5221, 9.4	8337, 11.4	10097, 11.4	10239, 12.3	10893, 13.6
Prior Valve Surgery / Procedure		5457, 1.4	745, 1.3	1057, 1.45	1373, 1.6	1178, 1.4	1104, 1.4
Prior PCI		154064, 40.5	19835, 35.6	28157, 38.6	34980, 39.4	34911, 41.9	36181, 45.2
Prior CABG		70942, 18.6	8540, 15.3	13109, 18.0	16230, 18.3	16855, 20.2	16208, 20.3
Currently on Dialysis		8298, 2.2	1221, 2.2	1783, 2.5	1816, 2.0	1663, 2.0	1815, 2.3
Cerebrovascular Disease		47072, 12.4	5811, 10.4	8740, 12.0	10920, 12.3	10508, 12.6	11093, 13.9
Peripheral Arterial Disease		48300, 12.7	5892, 10.6	8827, 12.1	11156, 12.6	10713, 12.9	11712, 14.6
Chronic Lung Disease		60486, 15.9	7140, 12.8	10219, 14.0	13067, 14.7	14174, 17.0	15886, 19.9
Diabetes Mellitus		135509, 35.6	18218. 32.7	25760, 35.3	30758, 34.6	29993, 36.0	30780, 38.5
PCI Indication							
	Immediate PCI for STEMI	53677, 14.1	9329, 16.7	11110, 15.2	13073, 14.7	10926, 13.1	9239, 11.6
	PCI for STEMI (Unstable, >12 hrs from symptom onset)	3217, 0.8	649, 1.2	572, 0.8	777, 0.9	641, 0.8	578, 0.7
	PCI for STEMI (Stable, > 12 hrs from symptom onset)	2210, 0.6	362, 0.7	431, 0.6	525, 0.6	439, 0.5	453, 0.6
	PCI for STEMI (Stable after successful full-dose thrombolysis)	1696, 0.5	348, 0.6	343, 0.5	359, 0.4	366, 0.4	280, 0.4
	Rescue PCI for STEMI (after failed full-dose lytics)	2047, 0.5	382, 0.7	446, 0.6	445, 0.5	434, 0.5	340, 0.4
	PCI for high risk Non-STEMI or unstable angina	171123, 44.9	24442, 43.9	32936, 45.2	41591, 46.8	37576, 45.1	34578, 43.2
	Staged PCI	24509, 6.4	2585, 4.6	4132, 5.7	5094, 5.7	6668, 8.0	6030, 7.5
	Other	122358, 32.1	17616, 31.6	22933, 31.5	26988, 30.4	26338, 31.6	28483, 35.6
CAD Presentation							
	No symptom, no angina	35325, 9.3	4642, 8.3	6374, 8.7	8195, 9.2	8536, 10.2	7578, 9.5
	Symptom unlikely to be ischemic	11931, 3.1	1489, 2.7	2199, 3.0	2935, 3.3	2750, 3.3	2558, 3.2
	Stable angina	65030, 17.1	9297, 16.7	12858, 17.6	14204, 16.0	14302, 17.2	14369, 18.0
	Unstable angina	138774, 36.4	17834, 32.0	25413, 34.8	32028, 36.0	31507, 37.8	31992, 40.0
	Non-STEMI	68816, 18.1	11681, 21.0	13521, 18.5	16736, 18.8	13877, 16.6	13001, 16.3
	ST-Elevation MI (STEMI) or equivalent	61036, 16	10774, 19.3	12572, 17.2	14776, 16.6	12426, 14.9	10488, 13.1
Coronary Artery Stenoses							
	0	21969, 6.4	2610, 5.1	3694, 5.6	5073, 6.4	4633, 6.1	5959, 8.4
	1	178870, 51.9	28113, 54.4	34661, 52.4	41736, 52.4	39093, 51.6	35267, 49.6
	2	90822, 26.4	13711, 26.5	17617, 26.6	20920, 26.3	19947, 26.3	18627, 26.2
	3	52732, 15.3	7293, 14.1	10169, 15.4	11857, 14.9	12135, 16.0	11278, 15.9
Presence of Proximal LAD Stenosis		97810, 28.2	14842, 28.3	18955, 28.7	22789, 28.3	20942, 27.6	20282, 28.3

**Abbreviations:** CABG = coronary artery bypass grafting; CAD = coronary artery disease; LAD = left anterior descending; MI = myocardial infarction; PCI = percutaneous coronary intervention; STEMI = ST elevation myocardial infarction

### Appropriateness of PCI

**Fig 3:**
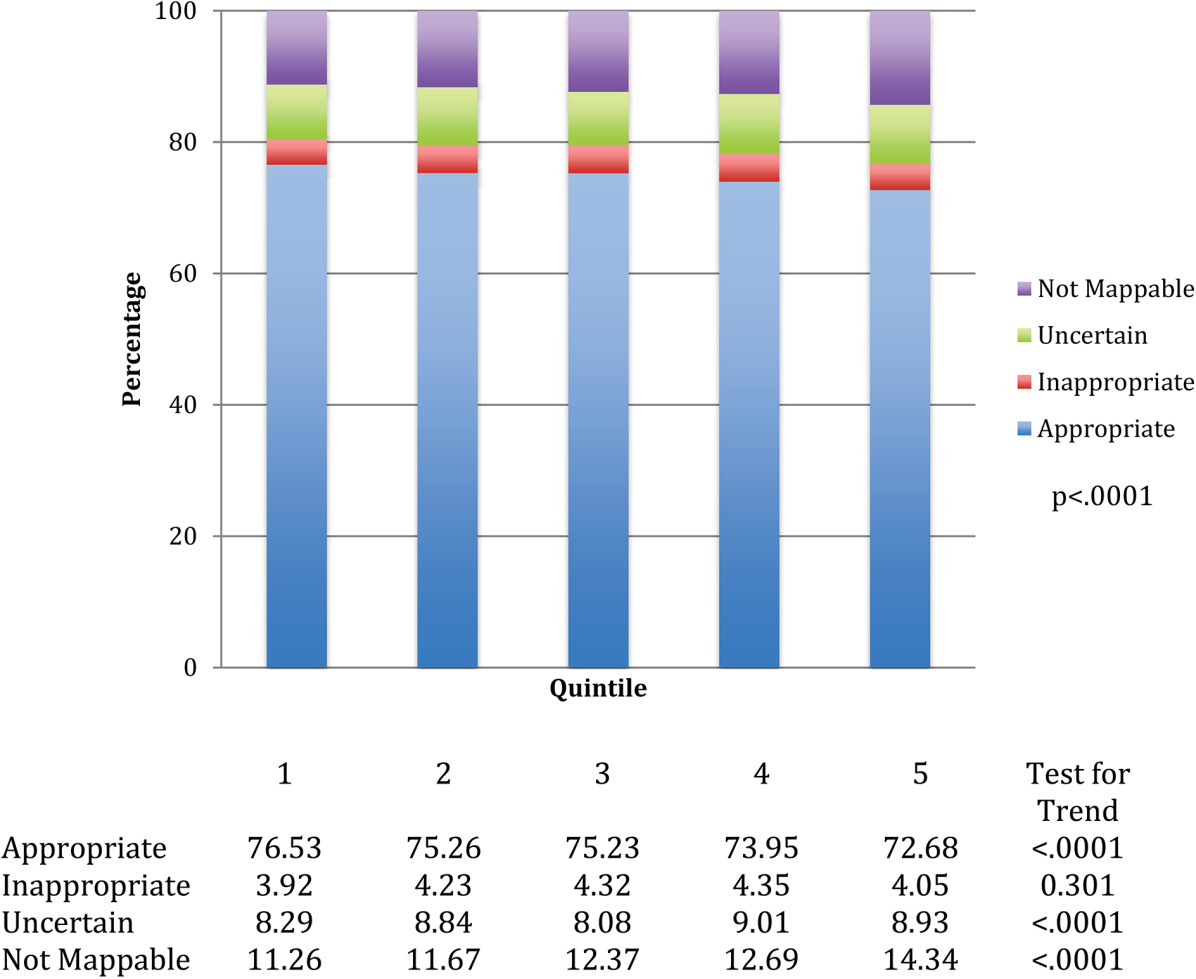
Distribution of PCI appropriateness across HRR quintiles for all PCIs. Shown is the application of the appropriate use criteria to quintiles of PCI utilization for all PCIs.

The rates for appropriate, inappropriate, uncertain, and not mappable for all PCIs in each quintile are displayed in **Fig 3**. Lower utilization areas had a higher rate of appropriate PCIs (Quintile 1, 76.5%, Quintile 5, 72.7%) while there is an increase in rates of unmappable PCIs in higher utilization regions (Quintile 1, 11.3%, Quintile 5, 14.3%). There was a smaller variation seen in the rates of inappropriate PCI (from 3.92% in the lowest volume quintile vs. 4.19% in the higher quintiles).

**Fig 4:**
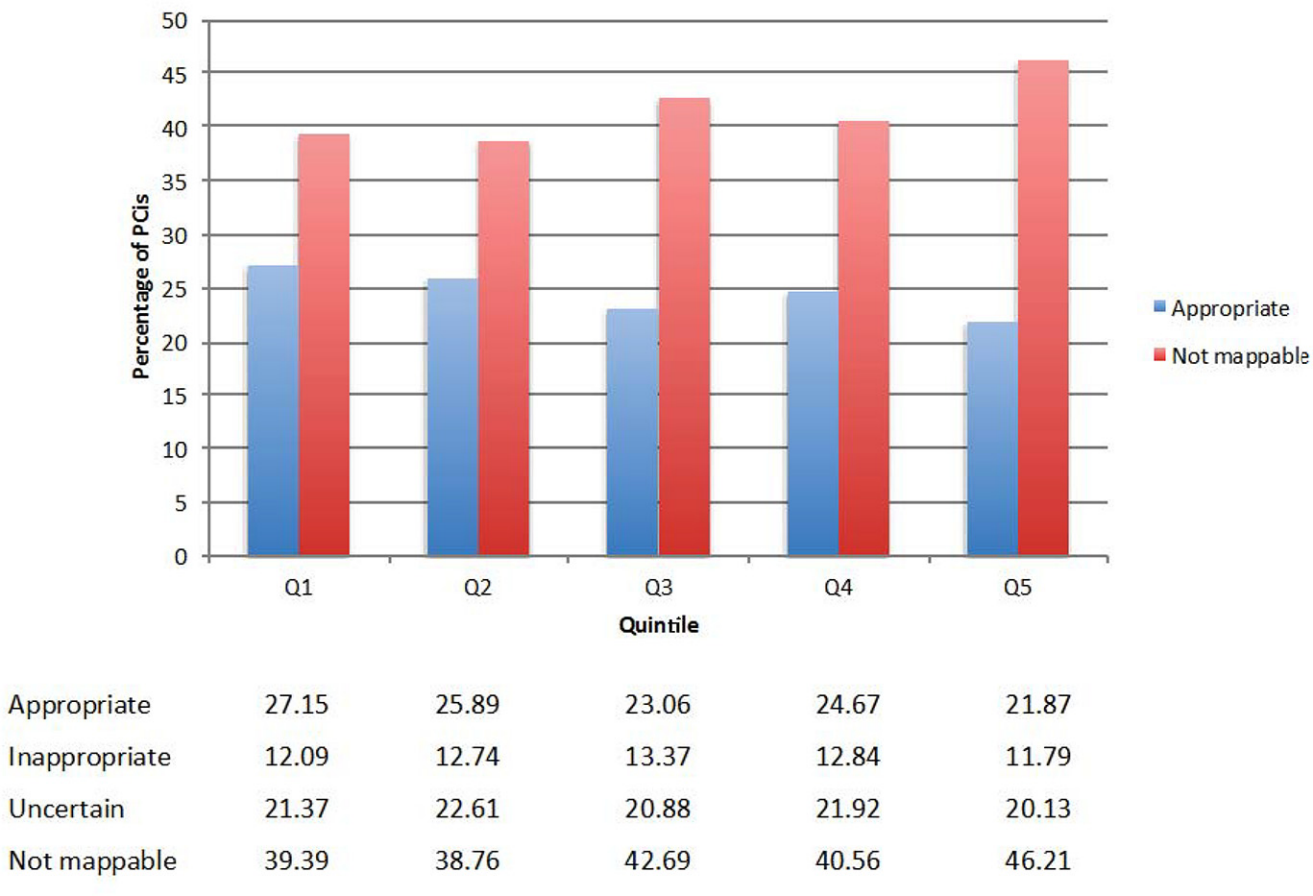
Appropriateness use criteria classification of non- acute PCI across HRR quintiles of PCI utilization. Shown is the application of appropriate use criteria to quintiles of PCI utilization for non-acute PCIs.

When stratified by clinical status, the rates of appropriate PCIs for acute indications was high (range 95.14%- 96.05%) across all the quintiles. However, the rates of appropriate PCIs for non-acute indications for each quintile varied across these regions and decreased from Quintile 1 to Quintile 5. Importantly, the rates of unmappable PCIs increased substantially from Quintile 1 to Quintile 5, with smaller variation in the rates of inappropriate PCI. With progressing from Quintile 1 to Quintile 5, the rates of appropriate PCI decreased from 27.15% to 21.87% while the ranges of inappropriate PCI were 11.79% to 12.74% and uncertain PCI were 20.13% to 22.61%, however the rates of unmappable PCI increased from 39.39% in Quintile 1 to 46.21% in Quintile 5 (**Fig 4**). A significant difference exists across the quintiles (p < .0001) for the entire cohort as well as among sub group of patients undergoing acute and non acute PCI. There was no difference in risk-adjusted mortality across quintiles of PCI utilization (**Table 4**).

**Table 4:** In-hospital mortality rates by quintile of PCI rate.

		Quintile 1	Quintile 2	Quintile 3	Quintile 4	Quintile 5
All PCIs						
	Adjusted mortality rate	1.35	1.34	1.28	1.32	1.38
Acute PCIs						
	Adjusted mortality rate	1.81	1.81	1.73	1.79	1.87
Non-acute PCIs						
	Adjusted mortality rate	0.25	0.22	0.21	0.21	0.24

**Abbreviations:** PCI = percutaneous coronary intervention

### Sensitivity Analysis

When we restricted the analysis to HRRs with 100% penetrance of the CathPCI Registry, there remained consistent variation in the proportion of acute and non-acute PCIs across utilization areas with lower utilization areas performing a higher percentage of acute cases. In Quintile 1, 73.27% of cases were for an acute indication compared to Quintile 5 where 66.76% of the PCIs were for ACS. (**S2 Fig**) The appropriateness of the cases progressively decreased from Quintile 1 to Quintile 5, due primarily to the greater use of PCI in ACS cases in the lower utilization regions. (**S3–S5 Figs**)

## Discussion

In this large, national registry, we evaluated the association between geographic variation in PCI rates and the appropriateness of these procedures using contemporary Appropriate Use Criteria. We observed differences among quintiles of PCI utilization as there was a decreasing percentage of appropriate use of PCIs and an increasing percentage of not mappable PCIs with increasing PCI utilization rates. As PCI utilization increases, the percentage of total case volume comprised of non-acute cases also increases, where the appropriateness of intervention is often lower and the rates of cases that are not mappable are higher compared to acute cases. Importantly, despite variation in the level of appropriateness, there was no clear influence on mortality.

Leape et al. examined geographic variation in rates of coronary angiography and its correlation with appropriateness in 1990. [14] The authors utilized data from 23 counties in one state and found that overall rate of coronary angiography (per 10,000 Medicare beneficiaries) varied from 13 to 158 and inappropriate use varied from 8 to 75%. The data suggested a positive correlation with use rate and inappropriateness. Furthermore, inappropriate use accounted for a 28% variance in rates. However, when the authors excluded one county, the amount of variance attributable to inappropriate use dropped and the correlation became insignificant. This led the authors to conclude that inappropriate use occurs in both low and high utilization areas and the objective should not merely be a reduction in the number of procedures performed, but elimination of those performed inappropriately in high or low utilization areas.

It is in this background and the findings of the current study, that the unmappable cases fall under greater scrutiny. Although a large proportion of unmappable cases make it difficult to precisely determine appropriateness, it is possible that some of the unmappable cases are discretionary and PCI may be able to be avoided. Unmappable PCIs include non-ACS presentations when a stress test was not performed or the results were not available. Recent data from Abdallah and colleagues suggests that patients who undergo elective PCI without a stress test do not have more severe symptoms or angiographic disease as compared with those undergoing PCI following stress tests. [15] This indirectly suggests that a proportion of the unmappable PCIs are likely inappropriate and that a substantial number of such PCIs in the high utilization areas could potentially be avoided.

This large variation in unmappable PCIs may provide a valuable target for quality improvement. Since there is little clinical downside to delaying PCI in the setting of stable CAD, ensuring appropriate risk stratification to better clarify the potential benefits of PCI seems reasonable. This process has been facilitated by the ready availability of online and downloadable applications supported by ACC and SCAI and is already a part of clinical work flow at many institutions across the country. [16]

Despite variation in the ratio of acute / non-acute cases across quintiles of PCI utilization and the decreasing rate of appropriate PCIs in higher utilization areas, rates of mortality were largely unaffected. Given that variations in appropriateness were mainly influenced by non-acute cases, this finding is not surprising since the non-acute patients are generally healthier and post PCI mortality or complications in this population are exceedingly uncommon. Our findings mirror prior work demonstrating that the proportion of inappropriate cases for a hospital is not related to outcomes such as mortality. [17]

### Study Limitations

The findings of this paper must be taken into context with the study’s limitations. Not all hospitals that perform PCIs within the United States participate in the CathPCI Registry. Additionally, the CathPCI Registry is a self-reported database and all cases do not undergo an audit. Furthermore, HRRs were excluded from the analysis given lack of penetrance of the CathPCI Registry within that HRR thereby excluding approximately 200,000 PCIs from analysis. This geographic-level analysis is also unable to tell us about individual patients and the appropriateness of their PCI. Some procedures that are considered inappropriate may indeed be appropriate when considering unique clinical features about the patient. Furthermore, the preferences of treatment choice by the patient were not assessed. Finally, the large proportion of unmappable cases highlighted not only reflects difficulty in applying the AUC to PCIs, but also to incomplete data sets. Of course, it is striking that there is a strong correlation between the number of unmappable PCIs and geographic variation in PCI use.

## Conclusions

Geographic regions that have low PCI rates have a higher proportion of PCIs performed for appropriate indications per AUC. Areas of high PCI utilization appear to perform more elective PCI and many of these procedures could not be mapped by the AUC. Our study findings support the need to explore the utility of routine application of AUC classification prior to elective PCI as a strategy to optimize utilization of PCI.

### Partners and Sponsors

CathPCI Registry is an initiative of the American College of Cardiology Foundation and The Society for Cardiovascular Angiography and Interventions.

## Supporting Information

S1 Fig PCI utilization across HRRs across the entire country. (DOCX)

S2 Fig Distribution of PCI appropriateness across quintiles of HRRs with 100% penetrance of the CathPCI Registry. (DOCX)

S3 Fig Appropriate use criteria categorization of PCIs across quintiles of HRRs with 100% penetrance of the CathPCI Registry. (DOCX)

S4 Fig Appropriate use of PCI by quintile for acute PCIs of HRRs with 100% penetrance of the CathPCI Registry. (DOCX)

S5 Fig Appropriate use of PCI by quintile for non-acute PCIs of HRRs with 100% penetrance of CathPCI Registry. (DOCX)

S1 Table Codes used to identify PCI. (DOCX)

## Abbreviations

PCI: percutaneous coronary intervention
NCDR: National Cardiovascular Disease Registry
AUC: appropriate use criteria
ACS: acute coronary syndrome
HRR: hospital referral region
