# Supplementary material for: Percutaneous Coronary Intervention Utilization and Appropriateness across the United States
Source: PLoS One. 2015 Sep 17;10(9):e0138251. doi: 10.1371/journal.pone.0138251 (PMC4575022; doi:10.1371/journal.pone.0138251)
Supplement: S1 Fig — (DOCX) [file pone.0138251.s001.docx]

**Supporting Figure 1:** **PCI utilization across HRRs across the entire country.**


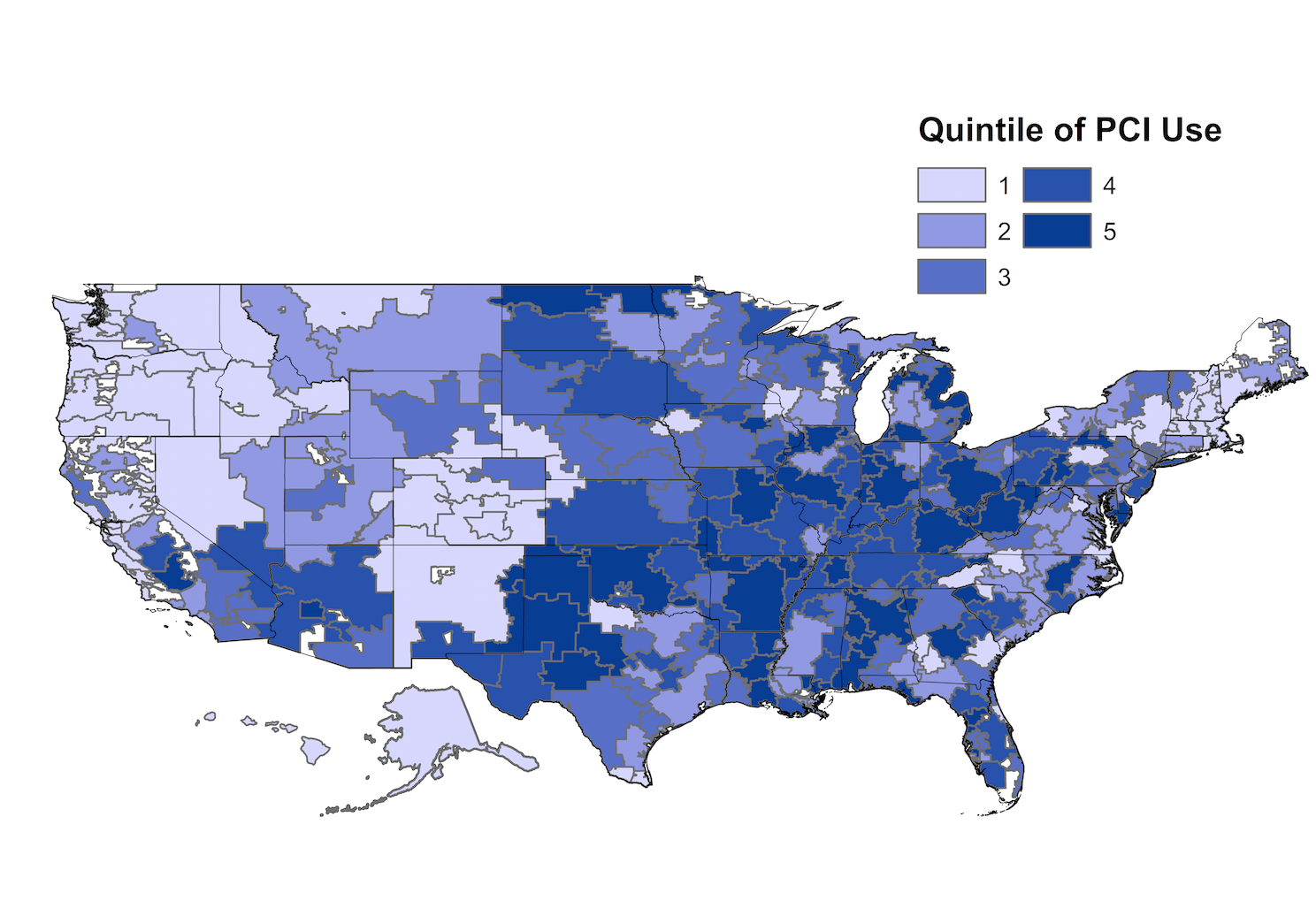


**Caption:** Shown is PCI utilization across all HRRs in the United States.

**Abbreviations:** PCI = percutaneous coronary intervention
