# Supplementary material for: Percutaneous Coronary Intervention Utilization and Appropriateness across the United States
Source: PLoS One. 2015 Sep 17;10(9):e0138251. doi: 10.1371/journal.pone.0138251 (PMC4575022; doi:10.1371/journal.pone.0138251)
Supplement: S3 Fig — (DOCX) [file pone.0138251.s003.docx]

**Supporting Figure 3: Appropriate use criteria categorization of PCI across quintiles of HRRs with 100% penetrance of CathPCI Registry**

**Caption:** Shown is the application of the appropriate use criteria to quintiles of PCI utilization in HRRs that have 100% penetrance of the CathPCI Registry.
