# Supplementary material for: Percutaneous Coronary Intervention Utilization and Appropriateness across the United States
Source: PLoS One. 2015 Sep 17;10(9):e0138251. doi: 10.1371/journal.pone.0138251 (PMC4575022; doi:10.1371/journal.pone.0138251)
Supplement: S4 Fig — (DOCX) [file pone.0138251.s004.docx]

**Supporting Figure 4: Appropriate use of PCI by quintile for acute PCIs of HRRs with 100% penetrance of the CathPCI Registry**

**Caption:** Shown is the application of appropriate use criteria to quintiles of PCI utilization for acute PCIs in HRRs with 100% penetrance of the CathPCI Registry.

**Abbreviations:** PCI = percutaneous coronary intervention
