# Supplementary material for: Percutaneous Coronary Intervention Utilization and Appropriateness across the United States
Source: PLoS One. 2015 Sep 17;10(9):e0138251. doi: 10.1371/journal.pone.0138251 (PMC4575022; doi:10.1371/journal.pone.0138251)
Supplement: S1 Table — (DOCX) [file pone.0138251.s006.docx]

**Supporting Table 1: Codes used to identify PCI**

ICD-9-CM

00.66- Percutaneous transluminal coronary angioplasty or coronary atherectomy

17.55-Transluminal coronary atherectomy

36.06-Insertion of non-drug-eluting coronary artery stent(s)

36.07-Insertion of drug-eluting coronary artery stent(s)

CPT

92973-Percutaneous transluminal coronary thrombectomy

92980-Coronary Stents [single vessel]

92981-Coronary Stents [each additional vessel]

92982-Coronary Balloon Angioplasty [single vessel]

92984-Coronary Balloon Angioplasty [each additional vessel]

92995-Percutaneous Atherectomy

92996-Percutaneous Atherectomy [each additional vessel]
